# Values, preferences, and informational needs of individuals living with ANCA-associated vasculitis: a systematic review

**DOI:** 10.1093/rap/rkag057

**Published:** 2026-06-12

**Authors:** Mats Junek, Seungwon Choi, Yuan Qi, Ali Sulaiman A Al-Magooshi, Gordon Guyatt, Lehana Thabane, Arielle Mendel, Nader Khalidi, Michael Walsh

**Affiliations:** Division of Rheumatology, St Joseph’s Healthcare Hamilton, Hamilton, Ontario, Canada; Department of Medicine, McMaster University, Hamilton, Ontario, Canada; Department of Medicine, Queen’s University, Kingston, Ontario, Canada; Division of Rheumatology, St Joseph’s Healthcare Hamilton, Hamilton, Ontario, Canada; Division of Nephrology, St Joseph’s Healthcare Hamilton, Hamilton, Ontario, Canada; Department of Health Research Methods, Evidence, and Impact, McMaster University, Hamilton, Ontario, Canada; Department of Health Research Methods, Evidence, and Impact, McMaster University, Hamilton, Ontario, Canada; Research Institute of St Joe’s Hamilton, St Joseph’s Healthcare Hamilton, Hamilton, Ontario, Canada; Lupus and Vasculitis Clinic, Division of Rheumatology, McGill University Health Centre, Montreal, Quebec, Canada; Division of Rheumatology, St Joseph’s Healthcare Hamilton, Hamilton, Ontario, Canada; Department of Medicine, McMaster University, Hamilton, Ontario, Canada; Division of Nephrology, St Joseph’s Healthcare Hamilton, Hamilton, Ontario, Canada

**Keywords:** vasculitis, systematic review, delivery of health care, quality of health care, quality of life

## Abstract

**Objectives:**

ANCA-associated vasculitis (AAV) is associated with a marked health burden and uncertainties across multiple treatment options. We aimed to complete a systematic review of previous research concerning individual values, preferences and informational needs in AAV to inform efforts to facilitate shared decision-making that can help manage this uncertainty.

**Methods:**

We conducted a qualitative systematic review with thematic synthesis of studies assessing values and preferences of individuals with AAV. We searched the CENTRAL, Medline, Embase, PsychInfo, HealthStar, CINAHL and three health decision databases for studies that assessed values, preferences, the utility of a decision aid and/or unmet informational needs for adults with AAV on 27 August 2024. The thematic synthesis approach guided our analysis. Risk of bias was assessed using the Critical Appraisal Skills Programme tool for qualitative studies and the Risk of Bias in Studies of Values and Utilities tool for quantitative studies.

**Results:**

We identified 1038 abstracts and included 18 studies. Seven qualitative studies included at least 177 individuals; the 11 quantitative studies included 1727 individuals. Three themes emerged: ‘living with AAV’, focusing on self-management needs and living within a supportive environment; ‘information exchange’, focusing on effective sharing of information and methods of information sharing; and ‘treatment of AAV’, focusing on preferences and experiences regarding the benefits and harms of therapy. We did not find any studies on values and preferences regarding therapies aside from glucocorticoids and plasma exchange.

**Conclusion:**

AAV affects many aspects of lives of individuals living with it, raising multiple issues around values, preferences and informational needs. Understanding these common concerns can improve care for individuals with AAV and facilitate shared decision-making.

Key messages

- Individuals with AAV have substantive heterogeneity in both disease and treatment values, preferences and experiences.
- Individuals with AAV report they have insufficient information about their disease.
- Addressing values, preferences and informational needs may facilitate shared decision-making and better disease outcomes.

## Introduction

ANCA-associated vasculitis (AAV) is a group of chronic, remitting and relapsing multisystem autoimmune diseases with potentially life- or organ-threatening manifestations. There are many effective therapies for AAV and each differs in efficacy, routes and frequencies of administration, monitoring requirements, availability/accessibility, potential side effects and risks, including infection [1–3]. These differences can create the potential for wide variability in treatment selection for AAV.

Shared decision-making (SDM) is recommended whenever there is more than one medically reasonable option for a patient’s care, and the choice depends on the patient’s personal values and preferences. SDM can improve adherence and individual satisfaction with care and can reduce decisional conflict [4, 5]. SDM is relevant to AAV given the number of treatment options available and their differences. To effectively engage in SDM, clinicians must understand the patient’s individual values, preferences and informational needs, defined as the beliefs, goals and expectations regarding one’s health and well-being; attitudes around treatment options; and the information required to understand what each treatment option encompasses, respectively [6]. Including these considerations ensures that the needs of the individual are centred in the SDM process.

To inform care and SDM, we undertook a systematic review to understand the values, preferences and informational needs of individuals living with AAV. These data can be used to help clinicians better engage the individuals they care for regarding their AAV.

## Methods

### Search strategy

We conducted a qualitative systematic review with thematic synthesis that was registered on PROSPERO (CRD420250574403). Using a comprehensive, preplanned search strategy, we searched CENTRAL, Medline, Embase, PsychInfo, HealthStar, CINAHL, International Network of Agencies for Health Technology Assessment, Ottawa Hospital Research Institute and Cochrane decision aids databases from inception to 27 August 2024 for articles that assessed specific values and preferences of individuals with AAV who were at least 18 years old (search strategy in Appendix A).

Our preplanned search strategy was initially designed to capture studies that assessed only treatment in AAV. However, early screening demonstrated the difficulty differentiating treatment-related from treatment-unrelated values and preferences through our search strategy. We therefore included values and preferences around any aspect of AAV including but not limited to the diagnosis, care environment, treatment and communication regarding care. Where we could not find the full text of a given study, we contacted authors to obtain original manuscripts; studies that were only available as conference abstracts were also included. We searched citations from included studies for other relevant studies. Studies were excluded if they only assessed the burden of disease or lived experiences with symptoms, if they included other diseases and AAV-specific data could not be extracted or if they did not assess one or more of individual values or preferences, the utility of a decision aid or unmet informational needs concerning disease or therapy.

### Data collection

Abstract screening, full-text screening and data abstraction were performed in duplicate by independent reviewers (M.J., S.C., M.Q., A.S.A.M.) using the above inclusion and exclusion criteria and a standardized data collection form. Systematic review software (www.covidence.org) was used for screening. Separate reporting forms were used for qualitative and quantitative studies. We extracted the year of publication, study design, sampling method, inclusion and exclusion criteria, participant demographics, value(s) and/or preference(s) being assessed, results and results of any subgroup analysis (see Appendix B for the standardized data collection form). For quantitative studies, information on regression or statistical analyses were gathered. We resolved disagreements through consensus.

We assessed the risk of bias in duplicate for each included study using the Risk of Bias in Studies of Values and Utilities tool for quantitative studies and the Critical Appraisal Skills Programme checklist for qualitative studies [7, 8].

### Analysis

We analysed data using thematic synthesis as outlined by Thomas and Harden [9]. Thematic synthesis identifies underlying common ideas, barriers, facilitators and initiatives using a flexible strategy that can include both quantitative and qualitative data. We first coded data in duplicate (M.J., S.C., M.Q., A.S.A.M.) from the findings of the studies, using direct quotes when available. Emphasis was placed on coding study results and participant quotes rather than the interpretations of the study authors. For quantitative studies, we used discrete information concerning results of analyses as codes (e.g. ‘in assessing informational needs at the time of diagnosis, information concerning the disease and its treatment were rated the highest priorities using a 100 point scale where a higher score indicated a higher priority’ [10]). We then assessed the complete set of extracted codes for each study against the primary text of the manuscript to ensure that no information was lost during the extraction process.

**Figure 1:**
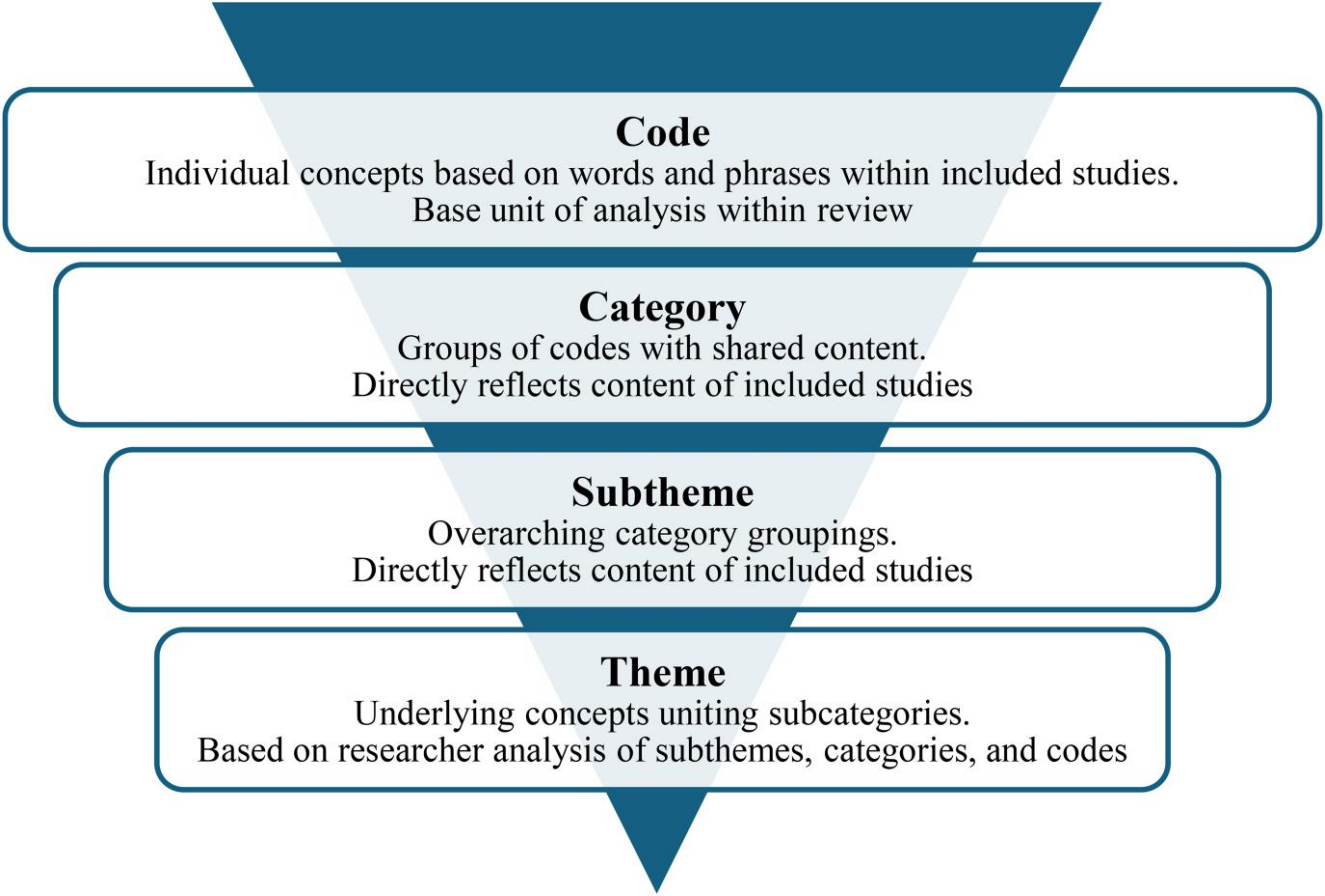
Organization of analytic data within the systematic review

A member of the research team (M.J.) organized the codes into categories of similar content (e.g. use of peer-to-peer support, utility of self-management strategies in AAV) (see Fig. 1) [9]. These categories were then assembled into broader subthemes (the above two categories fell into the subtheme of the need for self-care in AAV). Subthemes were finally organized into overarching themes that encompassed all relevant data but were not directly informed by included studies (e.g. self-care in AAV and the AAV care environment were encompassed by the theme of ‘living with AAV’). When completed, the thematic structure was assessed for face validity. We then repeated the process in reverse, starting with our themes and assessing for inclusion of subthemes, categories and then codes to ensure that the assembled thematic structure was both consistent with the data and inclusive of all findings. Finally, data extractors and a senior vasculitis researcher (M.W.) discussed the results to assess for completeness of coding, validity of thematic analysis and comprehensiveness of the thematic description of the results.

We summarized the systematic review process and described the characteristics of the selected studies. We summarized the thematic analysis and the risk of bias using narrative synthesis. No statistical analysis was performed; we did not use qualitative analytic software to assist the analysis. The reporting of this study is consistent with the Enhancing transparency in reporting the synthesis of qualitative research statement and the Preferred Reporting Items for Systematic Reviews and Meta-Analyses [11, 12].

## Results

### Search results

**Figure 2:**
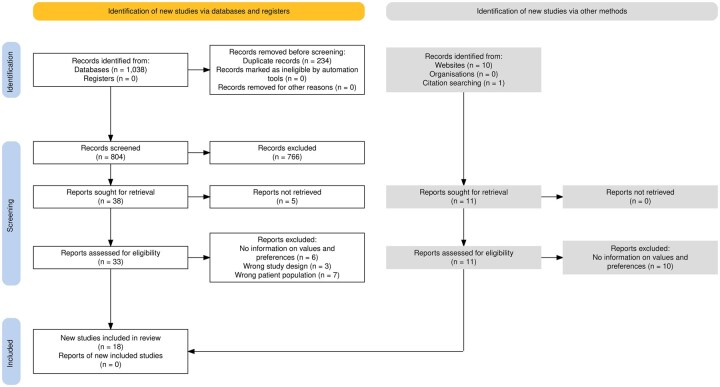
PRISMA flow diagram of study inclusion for the systematic review

The search yielded 1038 abstracts and 10 reports from separate searches in the International Network of Agencies for Health Technology Assessment, the Ottawa Hospital Research Institute database and Cochrane decision aids database, of which 17 studies were included. We identified one additional study during citation review from Granath *et al.*’s systematic review, for a final total of 18 included studies (see Fig. 2) [13–15]. All but 2 of the 18 included studies focused on values and preferences concerning various aspects of living with AAV. One of the non-dedicated studies assessed changes in values or preferences around AAV after a 1-day information seminar; the other included qualitative interviews to assess the feasibility, acceptability and benefits of participating in a randomized controlled trial of a physical exercise program to manage symptoms of AAV [16, 17].

Seven studies were qualitative studies and 11 were quantitative. Six studies collected qualitative data from 177 participants [17–22]. One study collected data from 746 comments produced by an unknown number of participants [23]. Qualitative studies, six of which included participants from the UK (see Table 1), focused on the experiences of being diagnosed and treated for AAV, the utility and acceptability of interventions for AAV, the experience of living with AAV and the pros and cons of the AAV care environment (Supplementary Tables S1 and S2). The 11 quantitative studies included 1727 participants, all with AAV. The majority were in Europe, the UK and the USA (Table 2) [10, 14, 16, 24–31]. Quantitative studies focused on participant experiences and informational needs around a diagnosis of vasculitis, preferences around the benefits and risks of treatments and aspects of AAV that are important for understanding a participant’s response to treatment (Supplementary Tables S3 and S4).

**Table 1:** Characteristics of individuals in included qualitative studies.

Study	Countries of participants	*n*	Male/female, *n*	Age (years)	Diagnosis, *n*	Disease duration (years)
Floyd *et al.* 2024 [18]	UK	19	10/9	Mean 66.6	19 AAV NOS	Mean 7.4
Harper *et al.* [17]	UK	43	25/18	<65 (*n* = 22), >65 (*n* = 21)	28 GPA, 11 MPA, 4 EGPA	
Mooney *et al.* 2012 [22]	UK	21	7/14	Median 60 (IQR 52–67)	14 GPA, 1 MPA, 6 EGPA	Median 9.5 (IQR 4.5–12.5)
Robson *et al.* 2017 [19]	USA, UK, Canada	51	24/27	<50 (*n* = 11), >50 (*n* = 40)	26 GPA, 7 MPA, 18 EGPA	34 <2 years and 17 >2 years from diagnosis or flare
Rutherford *et al.* 2018 [21]	Four unnamed European countries	33	11/22	<40 (*n* = 3), 40–70 (*n* = 25), >70 (*n* = 5)	20 GPA, 12 MPA, 1 EGPA	Median 3.5
Strobel *et al.* [23]	16 countries internationally	746^a^			EGPA	
Thorborg *et al.* [20]	Denmark	10	7/3	22–73	10 AAV NOS	Mean 2.5
Qualitative totals		177	84/93		88 GPA, 31 MPA, 29 EGPA, 29 AAV NOS	

a Number of online comments assessed rather than individuals.

NOS: not otherwise specified; GPA: granulomatosis with polyangiitis; MPA: microscopic polyangiitis; EGPA: eosinophilic granulomatosis with polyangiitis.

**Table 2:** Characteristics of individuals in the quantitative studies included in the systematic review.

Study	Countries of participants	*n*	Male/female, *n*	Age (years), mean (s.d.) or n (%)	Diagnosis	Disease duration (years)
Berg *et al.* [24]	USA	107	41/66		107 AAV NOS	
Brolin *et al.* [10]	Sweden	26	3/23	Mean 58.5 (s.d. 16.2)	26 AAV NOS	
Brolin *et al.* [25]	Sweden	17	8/9	20–66	15 GPA, 2 MPA	Mean 0.2
Collister *et al.* [26]	Canada, USA, UK	470	140/330	Mean 58.6 (s.d. 14.3)	334 GPA, 81 MPA, 55 AAV NOS	
Garbe *et al.* [16]	Germany	36	16/20	Mean 63.1 (s.d. 10.7)	36 AAV NOS	Mean 9.7 (s.d. 7.3)
Milman *et al.* [27]	USA, UK	99	38/61	Mean 56	67 GPA, 14 MPA, 18 EGPA	Mean 7
Mooney *et al.* [28]	USA, UK	587	287/300	Median 63	448 GPA, 34 MPA, 105 EGPA	66 <1, 521 >1
Quinn *et al.* [29]	Africa, Australia, Europe, North America	89	28/61	18–29 (*n* = 4), 30–49 (*n* = 16), 50–79 (*n* = 67), ≥80 (*n* = 2)	72 GPA, 17 MPA	<5 (*n* = 35), 6–10 (*n* = 27), 11–20 (*n* = 18), ≥21 (*n* = 9)
Thorpe *et al.* [30]		202	93/109	Mean 54.9 (s.d. 14.6)	145 GPA, 16 MPA, 10 EGPA, 31 RLV	Mean 6.3 (s.d. 5.9)
Wallace *et al.* [31]	USA	41	18/23	Mean 64.7	41 AAV NOS	
Yardimci *et al.* [14]	Canada	53	14/39	Mean 56.7 (s.d. 14.5)	53 GPA/MPA	Mean 9.9 (s.d. 7.3)
Quantitative totals		1727	686/1041		1081 GPA, 164 MPA, 133 EGPA, 318 AAV NOS, 31 RLV	

NOS: not otherwise specified; GPA: granulomatosis with polyangiitis; MPA: microscopic polyangiitis; EGPA: eosinophilic granulomatosis with polyangiitis; RLV: renal limited vasculitis.

### Risk of bias

Supplementary Tables S5 and S6 present results of the risk of bias for qualitative and quantitative summaries. The relationship between the participant and researcher was missing in qualitative studies in all but one qualitative study (Thorborg *et al.* [20]). All quantitative studies were vulnerable to results being shaped by sampling bias; however, 7 (63.6%) had low enough attrition and/or sufficient respondents to minimize this threat. One study had an extremely serious risk of bias [16].

### Coding and analysis

The initial coding of qualitative and quantitative studies yielded 117 separate codes considered potentially relevant to the research question. A total of 108 were relevant to values, preferences and needs in AAV. We excluded nine codes as they pertained to values and preferences of researchers rather than participants or to the emotional burden of AAV rather than a value or preference. We grouped the 108 remaining codes by common informational content into 17 categories. These categories mapped to six subthemes that assembled into three themes: living with AAV, information exchange and treatment of AAV (Tables 3 and 4). When we applied this thematic framework to the codes and primary data, we found they accurately reflected the data; no new codes, categories, subthemes or themes emerged.

**Table 3:** Summary of themes, subthemes and categories of information with definitions.

Theme	Subtheme	Category	Definition
Living with AAV	Ability to self-care for AAV	Self-management	Individuals value the ability to use self-care/self-referred (e.g. complementary medicine) strategies to manage their AAV
		Availability of peer-to-peer support	Individuals value sharing information with peers and finding supports for living with their disease
		Access to social/psychologic support	Individuals found that validation of their symptoms and having support persons was valuable with a diagnosis of AAV; however, the availability of psychological support was given variable priority
	AAV care environment	Professional communication in care	Individuals valued having a dedicated care team that understood their rare disease and could help navigate AAV
		Undesirability of misdiagnosis and care delays	Individuals value having a system that validates, investigates and can identify their symptoms in a timely fashion
		Availability of a dedicated/collaborative care environment	Individuals valued having a dedicated, supportive care staff for their disease, longitudinal relationships and receiving collaborative care about their disease
Information exchange	Diagnostic dilemmas and uncertainty	Need for information concerning the diagnosis	Individuals report getting information about their disease as a high priority throughout their disease journey
		Need for information concerning disease trajectory and outcomes	Individuals regularly want information about the trajectory of their disease, damage and how to assess for relapse
		Need for information concerning treatment	Individuals found information about treatment to be a high priority early in the diagnosis
		Mismatched patient/physician priorities	Individuals prioritized feeling better and their disease being under control whereas physicians prioritized disease remission
	Methods of information sharing	Need for multimodal information sharing	Individuals prefer receiving information in multiple forms, with written and oral communication most valued
		Need for repeated information	Individuals reported that they prioritized information about their disease both at diagnosis and during ongoing care
		Need for shared decision-making	Individuals expressed variable treatment preferences, recognized risks and benefits of therapies and valued having open and honest conversations with their clinical team concerning treatment options
Treatment of AAV	Benefits of treatment	Steroids improve symptoms of AAV	Individuals valued that steroids can quickly provide disease control in active AAV
		Treatment is effective in EGPA	Individuals report that they could get back to normal life when their EGPA was under control
	Harms of treatment	Adverse effects of glucocorticoids	Individuals reported the psychological changes, weight gain, sleep changes, facial changes and dorsal fat pad to be the most problematic side effects of being on glucocorticoids
		Desire to avoid adverse effects of treatment	Individuals placed high value in understanding the infection and serious adverse effect risks with any treatment of AAV. There are concerns about the health impacts of long-term treatment for AAV

**Table 4:** Themes and subthemes with example quotes supporting subthemes.

Theme	Subtheme	Category	Supporting data
Living with AAV	Self-care in AAV	Efficacy of self-management strategies	‘To think I couldn’t walk up from here to there without puffing. My neighbour used to call me Darth Vader, but I was like that, I was really breathless, but after a few weeks of doing this exercise all of a sudden I think “Hang on I haven’t gasped much today!” so it has helped my chest, because that’s where my weakness is, the GPA, it mostly affects my lungs, so that is so improved, it’s brilliant’! — P03 (female, 14 years diagnosed, intervention [24])
			Self-management considered the second most important aspects of educational needs on a scale of 0 to 100 where higher scores indicated more importance (76.4/100) [10]
	Care environment of AAV	Professional communication in care	‘The rheumatology helpline, I’ve found that really good; I would go to rheumatology; I would initially ask rheumatology because they have the expertise’. — P5. Patients with AAV clearly want to know more about their condition and to be able to access knowledgeable practitioners but GPs and other primary care staff need to know more about where to access such information [22])
			Participants expressed a preference for using a PROM as part of their clinical care and felt that completing it prior to clinic would be most helpful and facilitate discussions with healthcare professionals [18]
Information exchange	Diagnostic delays and uncertainty	Information concerning the diagnosis	All participants described a depth of fear and the trauma of suddenly being diagnosed with a rare and serious disease. Some patients reported not being given much information at all, which was particularly distressing because they had not heard of the disease before, so could not call upon any previous knowledge: ‘Well, first of all I didn’t quite understand, you know, I’d never heard of it or anything’. — P22. For such patients, this diagnosis of a rare condition was frightening, and their ignorance identified their need to know more. Further, the information is difficult to comprehend because of all the other things going on at this crucial time [of critical illness] [22]
			Information concerning the diagnosis was considered a priority for 90.1% of patients, similar to information concerning tests/treatment [28].
	Methods of information sharing	Need for multimodal information sharing	Patients in both the VUK and VCRC groups were highly desirous that information be provided by a doctor and supported by written material. In the VUK, the order of preference was doctor and written material, written material alone, doctor alone, Internet, group education, digital video disc (DVD), CD and 12-day course. In the VCRC group the order was Internet, doctor and written, doctor alone, written alone, education group, DVD, 12-day course and CD [28]
			Several participants wanted to be able to talk to an HCP about the disease. That kind of talk should take place where the participant and the HCP are sitting together and have the opportunity to ask clarifying questions. The participants expressed a need for information both orally and in writing, as expressed by one participant: ‘Maybe it could [be] a supplement, maybe it would have made me search less for information? Maybe it could have helped, right’? — P7 It may not be enough with oral information, because some patients stated they forgot the information, especially the information they received in the beginning when they received the diagnosis. They experienced being in shock and not able to consume the information [20]
Treatment of AAV	Benefits of treatment	Steroids are helpful in managing AAV	‘Well, I guess it was important that I was embarking on some treatment. You know, particularly with the high-dose steroids, you know, I really felt good’. — 71-year-old female, EGPA, USA ‘I came in here on Friday. I could not walk. I started the steroids on Saturday and on Sunday I was walking down the ward’. — 69-year-old male, GPA, UK [23]
			Patients reported that they were ‘thankful for the efficacy of GCs’ [37]
	Harms of treatment	Adverse effects of glucocorticoids	‘I have been reduced it (*sic*) steroid tablets but am concerned about all the side effects from the medications. I have started to get joint [pain] and particularly in the knees and elbow. I am taking several medications to combat steroid side effects. Please can anyone advise how you are coping and any advice on this problem’ [23]
			Moon facies, torso hump, weight gain, and insomnia rated the most severe side effects of glucocorticoids [14]

### Themes

The first theme, living with the burden of AAV, reflects how individuals with AAV often found challenges in managing their disease and how self-advocacy ensured they are understood and receive appropriate care. The first subtheme, self-care, encompasses the perceived benefits of self-management strategies, the value and validation provided by peer-to-peer support and/or engaging with other healthcare providers who understood their disease. The second subtheme focuses on the environments in which individuals with AAV receive care. Continuity of care and dedicated care teams were key qualities valued by individuals with AAV to feel understood and cared for [22].

The second theme, effective information exchange, underscores the frequent frustration individuals with AAV have with a lack of information around their disease and the inadequacy in its delivery. Evidence for this was seen in studies that directly assessed informational needs and in those that did not [10, 22, 23, 25, 28, 31]. The long journey to a diagnosis of AAV and being unwell at the time of the diagnosis often makes it challenging for individuals to understand the diagnosis at that time; information at follow-up was also limited. Many expressed a preference for both written and oral information provided to understand the diagnosis and the need to discuss information on multiple occasions [32]. Individuals with AAV also reported that participating in SDM allowed them to take ownership of their disease, as suggested by Quinn *et al.* [29], who found differences in individual-important and physician-important outcomes in AAV.

The final theme considered treatments for AAV. Individuals reported that treatment was generally effective and valued the marked improvement in disease symptoms. They also wished to avoid serious adverse effects, including infection. Both Yardimci *et al.* [14] and Robson *et al.* [19] directly assessed experiences, values and preferences with respect to glucocorticoids and Collister *et al.* [26] assessed the use of plasma exchange, however, there was minimal information concerning aspects of other treatments of AAV. Values and preferences around treatment were only formally evaluated around plasma exchange by Collister *et al.* [19]. Individuals with AAV reported that the psychological, skin and weight changes associated with glucocorticoid use were the most problematic and that finding the right dose could feel like an ‘experiment’ [19].

Finally, while not part of values and preferences, uncertainty was a recurrent, separate concept associated with all other themes. Individuals with AAV expressed anxiety concerning the events leading up to their diagnosis, a lack of understanding of the diagnosis and what was happening to their body, concerns regarding the degree of clinical improvement, frustration about side effects of glucocorticoids despite their effectiveness and ongoing concern of disease relapse preceded only by ambiguous signs or symptoms. Uncertainty regarding AAV (and subsequent associated anxiety) in day-to-day life was present in the themes of both living with AAV and information exchange despite mental health needs only being rated at moderate importance in one study [31].

## Discussion

Our systematic review found three central themes that characterized the values and preferences of individuals living with AAV: living with AAV requires self-advocacy and an effective care environment; understanding these rare diseases and their treatment is a constant need throughout their disease journey; and weighing AAV treatments is complex. Given that AAV is a rare disease with many areas of uncertainty, there are also many informational needs regarding the disease, its treatments and how to manage it within day-to-day life. These findings were seen in both qualitative and quantitative studies; however, they were better represented in qualitative studies, as quantitative studies more frequently assessed values and preferences in the context of a particular intervention or clinical question rather than across the entire AAV experience. Understanding these themes may help direct clinicians’ exploration of their patients’ values and preferences when considering treatment options.

These themes are not focused on concepts specific to the manifestations, treatments or outcomes of AAV. The subtheme most specific to AAV was the frequently documented fear and anxiety related to the unknown, particularly in the context of diagnostic ambiguity, delays and uncertain outcomes. The themes are also seen in other systemic autoimmune diseases such as RA [33, 34]. This suggests that individuals with multisystem autoimmune diseases have similar needs, values and preferences with common lenses, including understanding the risks and benefits of treatment, effective information exchange with others and personal lived experiences. Indeed, clinical decision aids designed to facilitate SDM around therapeutic de-escalation in RA have been designed using similar themes and have been found to help individuals navigate potential treatment de-escalation [35]. This suggests that researchers may be able to use shared findings from various diseases to facilitate SDM across other autoimmune diseases, a concept endorsed in recent guidelines for SLE [36].

We also found that while there are many therapies for AAV, there are only data concerning individual experiences with glucocorticoids (without considering dose or duration) and plasma exchange [19, 37, 38]. Rituximab, cyclophosphamide, azathioprine and other medications are all widely used in AAV [39, 40]. These are administered, monitored and experienced differently with varied efficacy and adverse effect profiles. Rituximab is currently the most effective medication in maintaining remission in AAV; it also cannot be reversed for months and carries risks of hypogammaglobulinemia and infections that may lead individuals to prefer other agents. Experiences, values and preferences in treatment may also shift over time—avoiding relapse may be an early priority but avoiding long-term adverse effects of treatment may be an increasing concern with longer treatment courses. This lack of data regarding non-glucocorticoid induction or maintenance therapies, longitudinal and/or dose-dependent exposure to glucocorticoids and how attitudes towards the treatment of AAV may change over time are an important gap in understanding lived experiences with AAV and are noted given the near-universal use of these therapies in the disease. Such data can be used to inform future patients regarding their choices in order to provide them with care consistent with their preferences.

Given the heterogeneity in both disease experience and treatments, understanding individual preferences may be of greater importance in those with AAV than other diseases [23, 29]. We are increasingly able to quantify the different risks of relapse, infection and other adverse effects across individuals with AAV [41, 42]. Each is differentially modified by the various agents used for induction and remission maintenance. Balancing efficacy, adverse effects and limited information regarding treatment in rare diseases involves considerable clinical uncertainty. Incorporating individual values, preferences and informational needs facilitates treatment decisions that balance these factors and minimize discomfort with residual uncertainty. Similar to work in RA, the use of clinical decision aids in SDM is one approach to facilitate this. In oncology, where treatment approaches parallel AAV rather than RA, clinical decision aids have been shown to reduce decisional conflict and increase patient satisfaction with care [4].

While the information needs concerning the disease and treatment are the highest priority at the time of diagnosis and decrease over time, information surrounding the diagnosis and its impact on day-to-day life remains a further ongoing need [25]. Similarly, the emotional/psychological burden of living with AAV, relapse, other long-term outcomes, treatment effects and need for support were all reported to be underappreciated by an individual’s treatment team [23, 37, 43, 44]. Individuals with AAV reported that these complex needs are best addressed in dedicated interdisciplinary vasculitis clinics, a finding seen in other multisystem rheumatic diseases [23, 45]. Measuring and addressing these issues during clinical care, however, can compete with the physician priority of monitoring the physical aspects of the disease. The use of AAV patient-reported outcomes provides a method for physicians to recognize some of these non-disease concerns; however, many of these instruments focus on facilitating a physician’s assessment of disease activity [18, 46, 47]. Understanding the needs, values and preferences of the individual with AAV continues to require directly engaging individuals around how their AAV has impacted their lives.

Our study uses a transparent and systematic method to gather and synthesize qualitative data into a cohesive framework. There are several limitations that must be considered in interpreting the findings. We included multiple databases in our search strategy to capture grey literature but qualitative information not published as research studies may not have been captured. There was also selection bias of participants—individuals who survived the onset of disease, who had an interest in research and the opportunity to be recruited and who were well enough to participate are overrepresented. Consequently, individuals who are most disadvantaged or most severely affected by AAV may be underrepresented, although individuals with severe disease were included [26]. We also found that existing research concerning values and preferences only addressed limited aspects of AAV with large gaps in our understanding of individual experiences with AAV. In addition, this study was a secondary qualitative analysis of reported data. Finally, while quantitative systematic reviews can abstract primary data, we were only able to abstract the data presented in the manuscripts. As such, some nuances and findings within the primary data that were not reported in the primary manuscripts but relevant to this analysis may have been missing.

This study demonstrates that individuals with AAV have a diversity of values and preferences across all aspects of their disease journey. Data concerning values and preferences around treatment of AAV are limited. They also have many informational needs across these rare diseases and their treatment that are often unmet. These findings collectively suggest that improved outcomes for individuals with AAV may be achieved through addressing these concerns in effective SDM without the need for new treatments.

## Supplementary Material

rkag057_Supplementary_Data

## Data Availability

The data underlying this article will be shared upon reasonable request to the corresponding author.
