# Supplementary material for: Values, preferences, and informational needs of individuals living with ANCA-associated vasculitis: a systematic review
Source: Rheumatol Adv Pract. 2026 Jun 12;10(3):rkag057. doi: 10.1093/rap/rkag057 (PMC13267788; doi:10.1093/rap/rkag057)
Supplement: rkag057_Supplementary_Data [file rkag057_supplementary_data.zip › VPSR Appendix A - Search Strategy.pdf]

DATE OF SEARCH -> 27 August 2024

Embase <1974 to 2024 August 26>

Ovid Healthstar <1966 to July 2024>

APA PsycInfo <1806 to August 2024 Week 4>

EBM Reviews - Cochrane Database of Systematic Reviews <2005 to August 21, 2024>

EBM Reviews - ACP Journal Club <1991 to July 2024>

EBM Reviews - Database of Abstracts of Reviews of Effects <1st Quarter 2016>

EBM Reviews - Cochrane Clinical Answers <August 2024>

EBM Reviews - Cochrane Central Register of Controlled Trials <July 2024>

EBM Reviews - Cochrane Methodology Register <3rd Quarter 2012>

EBM Reviews - Health Technology Assessment <4th Quarter 2016>

EBM Reviews - NHS Economic Evaluation Database <1st Quarter 2016>

Ovid MEDLINE(R) and Epub Ahead of Print, In-Process, In-Data-Review & Other Non-Indexed Citations, Daily and Versions <1946 to August 26, 2024>

1 exp Anti-Neutrophil Cytoplasmic Antibody-Associated Vasculitis/ 48934

2 ('ANCA-associated vasculitis' or 'ANCA associated vasculitis' or 'ANCA associated vasculiti*' or 'churg-strauss' or wegener or ((granulomatosis or microscopic) and polyangiitis) or ((ANCA or 'neutrophil cytoplasmic antibody' or 'antineutrophil cytoplasmic antibod*') and (vasculitis or glomerulonephritis or renal))).ab,ti. 46959

3 1 or 2 65906

4 exp Qualitative Research/ 347036

5 exp Data Collection/ 8376215

6 exp "Surveys and Questionnaires"/ 3583317

7 ('qualitative stud*' or survey* or questionnaire* or 'focus group*' or 'conjoint analysis' or 'discrete choice experiment*' or 'discrete choice' or 'rating task*' or 'ranking task*' or 'choice experiment*' or 'risk attitude*' or 'risk aversion' or 'standard gamble' or 'willingness to pay' or 'willingness-to-pay' or 'time trade*' or 'trade off*' or 'stated preference*' or 'contingent valuation' or 'choice experiment').tw. 6038451

8 4 or 5 or 6 or 712916271

9 exp decision support techniques/ 205209

10 exp decision support systems clinical/ 28921

11 exp decision trees/ 51087

12 exp Decision Making Computer Assisted/ 319738

13 (decision making or choice behavior).mp. and informed consent.sh. 28481

14 ((decision* or decid*) adj4 (support* or aid* or tool* or instrument* or technolog* or technique* or system* or program* or algorithm* or process* or method* or intervention* or material*)).tw. 486570

15 (decision adj (board* or guide* or counseling)).tw. 948

16 ((risk communication or risk assessment or risk information) adj4 (tool* or method*)).tw. 37626

17 (computer* adj2 decision making).tw. 1008

18 interactive health communication*.tw. 286

19 (interactive adj (internet or online or graphic* or booklet*)).tw. 4779

20 (interacti* adj4 tool*).tw. 20835

21 ((interactiv* or evidence based) adj3 (risk information or risk communication or risk presentation or risk graphic*)).tw. 188

22 shared decision making.tw. 56978

23 (informed adj (choice* or decision*)).tw. 56107

24 adaptive conjoint analys#s.tw. 263

25 9 or 10 or 11 or 12 or 13 or 14 or 15 or 16 or 17 or 18 or 19 or 20 or 21 or 22 or 23 or 24 1120957

26 exp Consumer Satisfaction/ 56134

27 exp Consumer Participation/ 103904

28 exp Patient Satisfaction/ 413020

29 exp "Attitude of Health Personnel"/ 567325

30 exp Health Knowledge, Attitudes, Practice/ 415593

31 exp "Delivery of Health Care"/ 7285170

32 exp Health Priorities/ 138822

33 exp Consensus/ 158376

34 (patient compliance or patient participation or patient satisfaction or treatment refusal or patient preference* or patient opinion* or patient belief* or patient concern* or patient perspective* or patient choice* or patient value* or patient priorit* or patient perception* or choice behavio* or patient consensus* or uncertaint* or discrete choice* or informational need*).tw. 864045

35 (dissent and dispute*).tw. 76

36 (utility or utilities).ti,ab. 965248

37 ((patient$ or participant$) adj3 (participation or satisfaction or perspective$ or compliance or preference$ or opinion$ or belief$ or concern$ or choice$ or value$ or priorit$ or perception$ or request$)).tw. 1182156

38 26 or 27 or 28 or 29 or 30 or 31 or 32 or 33 or 34 or 35 or 36 or 37 9918197

39 8 and 38 2565970

40 25 or 39 3566707

41 3 and 40 1038
