# Supplementary material for: Values, preferences, and informational needs of individuals living with ANCA-associated vasculitis: a systematic review
Source: Rheumatol Adv Pract. 2026 Jun 12;10(3):rkag057. doi: 10.1093/rap/rkag057 (PMC13267788; doi:10.1093/rap/rkag057)
Supplement: rkag057_Supplementary_Data [file rkag057_supplementary_data.zip › VPSR_Supplementary_tables_and_figures (1).docx]

**Supplementary tables and figures**

**Supplementary Table S1:** inclusion, exclusion, data collection, analysis, and research topics for qualitative studies

| **Study** | **Inclusion criteria** | **Exclusion criteria** | **Method of data Collection** | **Analysis** | **Research topic(s) assessed** |
| --- | --- | --- | --- | --- | --- |
| Floyd, 2024 | Diagnosis of AAV | - | Preliminary results with full analysis being completed | - | 1) The effects of AAV on everyday life and living, 2) the effects of treatment on everyday life and living, 3) the role of patients in health management and the patient experience, 4) a review of existing generic and disease specific patient reported outcome measure tools |
| Harper, 2021 | Age 18+, diagnosis of AAV, AAV in remission > 6 months (BVAS 0) and prednisone < 7.5 mg daily for 6 months, fatigue with MFI-20 > 14 | Unable to consent, unwilling to participate, comorbidities, unable to communicate in English | Focus group interviews of participants in a randomized clinical trial at end of trial, semi-structured telephone interviews with individuals who did not participate in trial | Framework method was used to perform thematic mapping done in duplicate | Experiences of participating in exercise program/intervention and impact on symptoms and psychological benefits |
| Mooney, 2012 | Adults with AAV or PAN and able to consent (Note: data from individuals with AAV able to be separately extracted) | Having concomitant severe medical problems or life-threatening disease; limited English and being aged less than 18 years. | Neutral, non-directional interview prompts and cues on the patient experience and impact on HRQoL of AAV and treatment. Continued until no new substantive themes and data saturation was achieved | The transcribed tapes were read and analysed by three  authors (J.M., F.P., N.S.) using the framework technique  (Ritchie and Spencer, 1994) | Informational needs of a new diagnosis and ongoing management of their condition |
| Robson, 2017 | Adults with AAV and able to consent | - | Neutral, non-directional interview prompts and cues on the patient experience and impact on HRQoL of AAV and treatment. Continued until no new substantive themes and data saturation was achieved | Data analyzed using Nvivo version 10 to assess themes related to glucocorticoids | Experiences of taking glucocorticoids while being treated for vasculitis |
| Rutherford, 2018 | Diagnosis of AAV | - | One-on-one interviews | Thematic analysis of the interview transcriptions done by one experienced researcher | Patient experience and views over AAV and treatment with a focus on treatment journey and unmet needs |
| Strobel, 2022 | Discussion of living with or caring for people with EGPA | Healthcare provider | Gathering post information in original language and translated to English | Results presented word-for word and as descriptive themes | Experiences of living with EGPA as posted on social media and communication with other individuals with EGPA around their lived experiences |
| Thorburg, 2022 | Recent diagnosis with AAV (within 5 years) | Dialysis, kidney-limited vasculitis, reduced cognitive ability, followed by other departments, not able to understand and speak Danish | Semi-structured qualitative individual interviews via telephone or facetime | Thematic analysis | Informational needs around diagnosis, indications for relapse, mental wellbeing, and emotional support |

MFI-20 = Multidimensional Fatigue Inventory 20; BVAS = Birmingham vasculitis activity score, HRQoL = Health-related quality of life; EGPA = eosinophilic granulomatosis with polyangiitis

**Supplementary Table S2:** Results from qualitative studies

| **Study** | **Results** |
| --- | --- |
| Floyd, 2024 | 1) Areas of HRQoL most important to participants: self-identity, social roles, fear of relapse, treatment burden. 2) Participants felt existing tools lacked consideration of some key issues, including overlooking positive factors that impact HRQoL, limitations regarding transient/inconsistent symptoms, as well as difficulty separating vasculitis from other conditions. 3) Participants expressed preference for using patient-related outcome measures as part of clinical care. 4) Areas acknowledged included: self-identity, social roles e.g. fulfilling caring responsibilities, fears of relapse and disease and treatment burden. Fifty-six deductive and inductive codes were identified which have been further categorized. Participants felt existing tools lacked consideration of some key issues. Scoring systems were considered to be negativity worded e.g. mild, moderate, severe, with the term ‘none’ being the only positive option. Participants reported that existing tools overlooked positive factors which impact HRQoL, such as symptom validation, close clinician monitoring and improvement with treatment. |
| Harper, 2021 | *Summary participant responses to participating in an exercise program for AAV*  1) Needed someone to encourage what to do and push me. Barriers included lack of time and distance from exercise program. 2) Fitbit made patient walk more, try for 10k steps and use step count as motivator for activity. 3) Coming to the exercise program gave me motivation to not give up. 4) It was helpful to get exercise techniques and feedback on exercises. Increased exercise tolerance. 5) The exercise program gave motivation for physical exercise. Able to mow lawn, garden and play with grandchildren. 6) Checking the Fitbit is motivational. |
| Mooney, 2012 | 1) Reaction to diagnosis: fear of a diagnosis of a rare disease and difficulty comprehending as multiple health problems lead to presentation and still sick when informed of diagnosis, didn't know questions to ask.  2) Understanding disease management: Patients were not given enough information; many were scared about cyclophosphamide for induction; understanding and managing drugs and side effects, but only information given was a simple print out and conflicting information was found online. Patients felt they needed to be assertive to have their disease information needs met.  3) Access to knowledgeable practitioners: only specialists understood disease, individuals value knowing people who understand the condition.  4) Provision of written information; use of simple language; conceptualization of communication as a two-way process and not just provider to patient; given time to absorb information. Review information when patient is well.  5) Want written information to have a sense of control and how to manage their diseases.  6) Appreciate a rheumatology help line and people to contact, as there was a sense of isolation from other people .who are not aware of the disease |
| Robson, 2017 | 1) Feel better getting treated after lots of investigations, feels things are being fixed. Flares on withdrawal and glucocorticoid reduction is an 'experiment'.  2) Impact of glucocorticoids on self appearance, comments from others, increased appetite, diabetes, skin changes, sleep disturbance, change in muscle strength, and emotional symptoms.  3) Balance of short-term benefit vs long term effects of steroids. |
| Rutherford, 2018 | 1) Long journey to diagnosis leading to long lasting psychological damage, worsened with treatment burden following diagnosis. 2) Worse patient experience with sudden onset or misdiagnosis, low empathy by professionals and low understanding of their needs. 3) Patients wish to understand their future, duration of therapy, and when to expect return to normalcy 4) Patients have low awareness of how their response is assessed clinically and scales used by clinicians. To patients, what is important is "feeling better" and going home. 5) Low involvement in treatment decisions over glucocorticoids and immunosuppression. 6) Major side effects of glucocorticoids impair HRQoL, satisfaction, and functional status. |
| Strobel, 2022 | 1) Personal experience is the most common theme. Many posts shared challenges and coping methods in relation to EGPA.  2) 38% of posts on EGPA looked for advice on symptom management and replies frequently contained advice and encouragement.  3) The most frequently mentioned EGPA symptoms were asthma, neuropathy, sinus issues, pain, and fatigue. Posts also mentioned skin, digestive, and musculoskeletal issues on a less frequent basis.  4) Efficacy and safety of steroids and biologics for EGPA and success stories. Also discussed potential negative effects of steroids.  5) 33% of comments from EGPA patients were related to diagnosis.  6) Feelings of control or lack of control.  7) Lack of disease awareness in both physicians and non-physicians  8) Negative experiences with healthcare providers were expressed in 75% of comments for EGPA: healthcare providers overlooking symptoms; diagnostic delays; incorrect diagnosis; no diagnosis; multiple referrals with no progress towards diagnosis; difficulty accessing treatments.  9) Feelings of vulnerability and invisibility during the COVID-19 pandemic.  10) Diagnosis and treatment of EGPA can incur significant costs in some countries. |
| Thorburg, 2022 | 1) Need for oral and written information: Both were needed and contributed differently, informed at the beginning but later missing 2) Need for information about living with the disease: symptoms, impact on everyday life, and living with disease. 3) Need for information on symptoms and relapse: feeling of disease as 'invisible' and highlighting symptoms of relapse. 4) Psychological support for information about the disease - caregivers/support when getting and interpreting diagnosis, awareness of mental health impacts. 5) Need for peer sharing of information - desire to have a friend/someone to go through experiences with. |

HRQOL = Health-related quality of life; EGPA = eosinophilic granulomatosis with polyangiitis

**Supplementary Table S3:** inclusion, exclusion, data collection, analysis, and research topics for qualitative studies

| **Study** | **Inclusion Criteria** | **Exclusion criteria** | **Method of data Collection** | **Analysis** | **Research topic(s) assessed** |
| --- | --- | --- | --- | --- | --- |
| Berg, 2013 | Diagnosis of AAV | - | Structured CAM questionnaires were administered at medical appointments | Descriptive statistics | Patient experiences, perceptions and values 1) Which CAM practices were used 2) Which CAM practices were used to improve well-being 3) Which CAM practices were helpful |
| Brolin, 2022 | Diagnosis of AAV, aged 18+, fluent in Swedish | Cognitive impairment, poor literacy | Administration of ENAT forms to consenting participants | Descriptive statistics | Educations needs seven domains (‘Managing pain’,  ‘Movement’, ‘Feelings’, ‘Disease process’, ‘Treatment’, ‘Self management’ and, ‘Support systems’) |
| Brolin, 2023 | AAV patient, over 18 years old, literate in Sweden | Cognitive impairment interfering with literate capabilities | Questionnaires given at around time of diagnosis and 24 months later | Descriptive statistics | ENAT questionnaire contained items from 7 domains (managing pain, movement, feelings, disease process, treatment, self-management and support) |
| Collister, 2023 | English speaking adults with self-reported clinical diagnosis of granulomatosis with polyangiitis or microscopic polyangiitis | Diagnosis of eosinophilic granulomatosis with polyangiitis | Online patient survey | Multilevel multivariable logistic regression models | Patient preferences concerning receipt of plasma exchange. Provided general information about plasma exchange. Then participants chose or declined plasma exchange treatment for different scenarios for which 1 year risk of kidney failure and serious infection with or without plasma exchange were given |
| Garbe, 2023 | German-speaking AAV patients between the ages of 18 and 90 years | High disease activity or severe concomitant disease precluding seminar attendance, and severe mental or cognitive impairment impeding knowledge transfer | Multiple choice test, questionnaire | Descriptive statistics | Patient preferences, perceptions, experiences and values  1) Need for disease-specific information  2) Cognitive and emotional representation of the AAV disease  3) Disease-relevant behaviour, level of present knowledge, importance of various disease-related topics  4) Nutritional preferences  5) Medication adherence |
| Milman, 2019 | Patients with AAV | Other types of vasculitis | Self-administered, questionnaire which was given in the symposia | Descriptive statistics | 1) Identify key areas of impact: discover which aspects of health, functioning and daily affected from their disease 2) Using a structural framework for analysis to systematically categorize these aspects 3) Compare patients priorities vs experts priorities |
| Mooney, 2014 | Members of Vasculitis United Kingdom Charity and the Vasculitis Patient-powered Research Network with a self-declared diagnosis of AAV | Respondents declaring other types of vasculitis | Survey designed using informational needs of information with cancer based on TINQ-BC for breast cancer. Focuses on disease, investigations, treatments, physical, and psychosocial impact, adapted as the Vasculitis Informational Needs Questionnaire (VINQ) | Descriptive statistics, Cronbach's alpha | Informational needs using quantitative metrics  1) Disease  2) Investigations  3) Treatment  4) Physical  5) Psychosocial |
| Quinn, 2022 | Diagnosis of AAV |  | Three-round online Delphi survey and a fourth ranking round to rank items within a domain | Descriptive statistics | Items considered essential by both patients and physicians to measure treatment response including disease activity, mortality, patient-reported outcomes, and disease-specific outcomes |
| Thorpe, 2008 | ≥18 years old, able to read and write in English, diagnosis of AAV | Diagnosis of Goodpasture's and temporal arteritis | Self-administered, mailed questionnaire | Descriptive statistics and regression analyses | Patient perceptions, experiences and values measured using:  1) 8 Self-management behaviors via Vasculitis Self-Management Scale (VSMS)  2) Obtaining specific barriers to self-management via checklist  3) Rating perceived difficulty of barriers  4) Social desirability  5) Patient demographics |
| Wallace, 2022 | Patients with AAV, IgG4-related disease or systemic sclerosis | - | Online survey or survey via phone/mail | Pearson's correlation coefficient and Cochran-Mantel-Hanszel test used to assess correlations | Health needs associated with uncertainty surrounding systemic rheumatic disease |
| Yardimci, 2023 | Individuals >18 with a diagnosis of vasculitis | - | Survey with questions on demographics, glucocorticoid use, glucocorticoid side effects, severity of side effects, most important side effect, and interest in switching from prednisone to an alternative | Descriptive statistics, assessed normality and Chi2 for microscopic polyangiitis vs granulomatosis with polyangiitis vs other vasculitis | Ranking side effects with glucocorticoid use; assessing knowledge and preferences around glucocorticoid alternatives |

CAM = Complementary and alternative medicines; ENAT = Educational Needs Activity Tool; TINQ-BC = Toronto Information Needs Questionnaire for Breast Cancer

**Supplementary Table S4:** Results of quantitative studies

| **Study** | **Outcome** | **Response format** | **Result** |
| --- | --- | --- | --- |
| Berg, 2013 | Perceptions on complementary and alternative medicine (CAM) treatments and self-help practices | Not stated | 81% had CAM practice, which included: prayer (n=68, 64%), exercise promotion (n=29, 27%), massage therapy (n=20, 19%), chiropractic services (n=14, 13%), acupuncture (n=7,7%)  Reasons for use was to improve well-being: Prayer (36/68, 53%), exercise promotion (25/29, 86%), massage therapy (14/20, 70%), relaxation techniques (13/22, 59%).  Modalities used: mind (n=30, 28%), mind-body (n=15, 14%).  24% of CAM users said that their doctor talked about CAM.  88% were comfortable sharing their CAM practices with their doctors.  48% of CAM users would recommend CAM to other AAV patients. |
| Brolin, 2022 | BVAS score and ENAT which is consisted of 39 questions covering 7 domains: managing pain, movement, feelings, disease process, treatment, self-management, and support | Mean % of domain score where 0 = lowest importance and 100 = highest importance presented as mean with standard deviation | 1) Managing Pain: 50.4 (32.0) 2) Movement: 49.7 (34.4) 3) Feelings: 71.9 (29.5) 4) Disease Process: 84.0 (20.0) 5) Treatments: 74.3 (28.6) 6) Self-management: 76.6 (22.9) 7) Support Systems: 59.7 (28.0) 8) Total ENAT score: 65.7 (23.3) |
| Brolin, 2023 | BVAS score and ENAT which is consisted of 39 questions covering 7 domains: managing pain, movement, feelings, disease process, treatment, self-management, and support | Mean % of domain score where 0 = lowest importance and 100 = highest importance presented as MSD and using paired T-tests | \| Domain \| Score at 0 months (%) \| Score at 24 months (%) \| Difference, P value \| \| --- \| --- \| --- \| --- \| \| Managing Pain \| 10.1 (56.1) \| 10.3 (57.2) \| 0.2, 0.89 \| \| Movement \| 8.5 (56.6) \| 7.5 (50) \| -1, 0.35 \| \| Feelings \| 8.4 (70) \| 7.3 (60.3) \| -1.1, 0.31 \| \| Disease Process \| 17.8 (84.7) \| 16.5 (78.6) \| -1.3, 0.25 \| \| Treatments \| 16.8 (80) \| 12.1 (57.6) \| -4.7, 0.005 \| \| Self-Help \| 15.4 (85.5) \| 12.9 (71.7) \| -2.5, 0.03 \| \| Support systems \| 6.9 (57.5) \| 6.3 (52.5) \| -0.6, 0.43 \| \| Total \| 83.5 (71.3) \| 72.6 (62) \| -10.9, 0.04 \| |
| Collister, 2023 | Patient preferences concerning the use of plasma exchange | 1) Yes or no to choosing treatment with plasma exchange in 5 scenarios  2) To evaluate the association between the decision to receive treatment with plasma exchange | 1) Scenario 1 (Creatinine [Cr] = 150): 54.9% chose plasma exchange, Scenario 2 (Cr = 250): 56.0%, Scenario 3 (Cr = 350): 63.4%, Scenario 4 (Cr = 450): 61.1%, Scenario 5 (Cr = 550): 57.9%  2) Results of multilevel multivariable logistic regression model  Younger age: OR 0.98 (CI 0.96-0.99 per 1 year increase)  Male sex OR 1.25 (0.73-2.15)  Country (reference Canada): UK OR 2.61 (1.09-6.22), USA OR 0.98 (0.48-1.99), Other OR 0.74 (0.20-2.74)  Previous dialysis: OR 2.70 (1.12-6.52)  Previous plasma exchange: OR 5.62 (2.72-11.61)  Previous infection OR 1.14 (0.70-1.85)  Scenario 2 (Cr 250) OR 1.08 (0.76-1.54)  Scenario 3 (Cr 350) OR 1.93 (1.35-2.77)  Scenario 4 (Cr 450) OR 1.61 (1.13-2.29)  Scenario 5 (Cr 550) OR 1.26 (0.88-1.79) |
| Garbe, 2023 | Need for information, disease-relevant behaviour pre- and post- one day seminar | Ordinal scales | Adoption of AHEI recommendations (before intervention %, after %):  Prefer fruits/vegetables (97.2, 96.8)  Prefer wholegrain (79.4, 87.1)  Prefer vegetable oils (80.0, 89.7)  Prefer fish (63.6, 83.3)  Moderate alcohol consumption (25, 26.7)  Avoid candy/sugar-containing drinks (63.9, 64.5; )  Avoid red meat (22.2, 25.8)  Avoid high salt foods (58.3, 71.0)  Avoid saturated fats (50, 46.7)  Percentage declaring "no help need" or "sufficient help available" on various disease-related topics (before intervention %, after %):  Disease specific information (34.2, 70.0)  Career and private security (63.9, 62.1)  Physician-patient relations (71.1, 76.7)  Social support (71.4, 66.7)  Psychological stress (62.9, 72.4)  Daily life (71.1, 79.3)  Physical limitations (71.4, 81.5) |
| Milman, 2019 | Compare patient vs clinician priorities from ICF framework in understanding how vasculitis affects their lives | Ordinal scales of relevance of ICF item to vasculitis | 24 ICF categories under body function, 6 under body structures and 4 under activities and participation were rated by more than 30% of participants as contributing to at least moderate impairment  4 ICF categories under products and technology, 5 under supports and relationships, 4 under attitudes and 6 under other environmental factors were rated by more than 30% of participants as being at least moderately relevant to their health.  3 ICF categories under demographics, 3 under personal history and biography, 5 under one's experience of vasculitis and 5 under personality, habits and patterns of behaviour were rated by more than 30% of participants as being at least moderately relevant to their health. |
| Mooney, 2014 | Informational needs | Adapted version of the Toronto information needs questionnaire into the Vasculitis information needs questionnaire with 5 point ordinal responses, separate questions concerning preferred methods of sourcing information | Domain, mean %: Disease, 90.1, Tests, 90.4, Treatment, 92.0, Physical, 86.0, Psychological, 59.0.  No difference in the response patterns between the VUK and VCRC cohorts regarding information needs, and no difference by gender, age, disease duration or self-report disease sub-type.  Preferred source of information  VUK: doctor and written material, written alone, doctor alone, Internet, group education, digital video disc (DVD), CD and 1-2 day course.  VCRC: Internet, doctor and written material, doctor alone, written alone, education group, DVD, 1-2 day course and CD. |
| Quinn, 2022 | Assessment of items/measured and their relevance to understanding outcomes in AAV | Scale of 1-9 | Number of physicians and patients who found a given item as highly important (ranked 7 or higher) in measuring response to treatment in trials of AAV   \| Item \| MD Responses \| % ranked as important \| Patient Responses \| % ranked as important \| \| --- \| --- \| --- \| --- \| --- \| \| Improved fatigue \| 176 \| 14% \| 89 \| 73% \| \| Improved pain \| 176 \| 28% \| 89 \| 74% \| \| Improved patient global assessment \| 182 \| 73% \| 107 \| 72% \| \| Improved HRQoL measures \| 176 \| 76% \| 89 \| 85% \| \| BVAS of 0 \| 176 \| 95% \| 89 \| 60% \| \| BVAS ≤1 \| 176 \| 95% \| 89 \| 62% \| \| >50% reduction in BVAS \| 176 \| 72% \| 89 \| 66% \| \| Improved kidney function (eGFR) \| 181 \| 96% \| 104 \| 83% \| \| No development of ESKD \| 181 \| 97% \| 104 \| 97% \| \| Ability to discontinue dialysis \| 176 \| 84% \| 89 \| 63% \| \| Resolution of hematuria urinalysis \| 176 \| 43% \| 89 \| 70% \| \| Resolution of proteinuria on urinalysis \| 176 \| 51% \| 89 \| 73% \| \| No new/worse major organ involvement \| 181 \| 96% \| 103 \| 95% \| \| Improved physician global assessment \| 181 \| 80% \| 96 \| 80% \| \| No rise in acute phase reactants \| 176 \| 27% \| 89 \| 71% \| \| Survival \| 182 \| 100% \| 109 \| 97% \| \| No new major organ damage \| 170 \| 94% \| 101 \| 97% \| \| No new non-major organ damage \| 176 \| 81% \| 89 \| 84% \| \| Severe medication-related adverse events \| 171 \| 94% \| 95 \| 91% \| \| Severe infections \| 176 \| 98% \| 89 \| 90% \| |
| Thorpe, 2008 | Patient perceptions, experiences, and values in vasculitis self management | Likert scales of importance for 8 self-management behaviours | \| Behaviour \| Frequency performed (SD)* \| Perceived difficulty** \| Perceived importance** \| \| --- \| --- \| --- \| --- \| \| Medication adherence \| 4.5 (0.5) \| 1.5 (1.0) \| 5.9 (0.4) \| \| Adherence to recommended health services \| 4.6 (0.6) \| 2.0 (1.4) \| 5.7 (0.7) \| \| Infection avoidance adherence \| 3.8 (0.8) \| 1.8 (1.3) \| 5.7 (0.6) \| \| Diet adherence \| 3.6 (0.8) \| 3.0 (1.5) \| 4.8 (1.2) \| \| Exercise adherence \| 3.1 (1.0) \| 3.2 (1.5) \| 4.7 (1.2) \| \| Symptom monitoring \| 3.8 (1.0) \| 2.0 (1.3) \| 5.1 (1.1) \| \| Adjusting activities \| 3.4 (0.7) \| 3.0 (1.6) \| 4.8 (1.3) \| \| Reporting Symptoms \| 3.2 (1.1) \| 2.4 (1.5) \| 4.8 (1.4) \| \| Reporting side effects \| 3.2 (1.1) \| 2.2 (1.5) \| 4.7 (1.5) \|   *Likert scale of 1-5 with higher scores indicating higher frequency; **Likert scale 1-6 with higher scores indicating higher difficulty/importance |
| Wallace, 2022 | Health needs associated with uncertainty surrounding diagnosis of systemic rheumatic diseases | Mishel Uncertainty in illness scale (MUIS), PHQ scale, GAD, Sickness impact profile, survey of psychosocial needs, and text responses | Stratified by disease activity into in remission/not in remission/ unknown, median [IQR]):  MUIS: 53 [43,56] / 56 [51,74] / 66 [59, 67] p=0.02  PHQ8: 2 [2,7] / 8 [5,19] / 4 [1,5] p=0.01  GAD7: 2[0,6] / 7 [4,10] / 5 [2,6] p=0.07  SIP: 1[0,2] / 5 [1,10] / 6 [0,8] p=0.06  No differences seen when stratified by sex.  Correlation coefficient between scales  MUIS and PHQ8: 0.56 (p=0.0001)  MUIS and GAD7: 0.45 (p=0.003)  MUIS and SIP: 0.57 (0.0001)  Needs identified by AAV patients, n (%)  1. Managing physical symptoms: 18 (43.9)  2. Coping with emotional concerns: 11 (26.8)  3. Managing social concerns/lack of support: 4 (9.8)  4. Managing sexual and reproductive concerns: 2 (3.8)  5. Finding resources: 4 (9.8)  6. Other: 4 (9.8)  7. Missing or none: 6 (14.6) |
| Yardimci, 2023 | Adverse effects of glucocorticoids (GC) and information around glucocorticoid alternatives | Numeric, Likert, and free responses | Mean duration of treatment with GC: 62.7+/-83.7 months.  All respondents had at least 1 GC related SE, 67% had more than 11 SE. Most common SE was mood change (95.8%), insomnia and weight gain (93.8%), and body disfiguration (91.7%). Body disfiguration was ranked the highest in severity score of 7.7+/-3.3/ 10, then weight gain at 7.5 +/-2.9/10, insomnia at 7.2+/-2.7/10, then decreased QoL at 7.2 +/-2.6/10.  43 respondents (44.3%) stated awareness of possible alternatives to prednisone. 56.5% of patients with GPA/MPA knew about avacopan, but only 1 used it before. A total of 66 (68%) stated they would prefer to be "one of the first patients outside of any study, to take a very new medication such as avacopan instead of going back on prednisone if they had a vasculitis flare." |

**Supplementary Table S5:** Risk of bias summary qualitative studies

|  | Floyd 2024 | Harper 2021 | Mooney 2012 | Robson 2018 | Rutherford 2019 | Strobel 2022 | Thorberg 2022 |
| --- | --- | --- | --- | --- | --- | --- | --- |
| Was there a clear statement of the aims of the research? | **Yes** | **Yes** | **Yes** | **Yes** | **Yes** | **Yes** | **Yes** |
| Is a qualitative methodology appropriate? | **Yes** | **Probably Yes** | **Yes** | **Yes** | **Yes** | **Yes** | **Yes** |
| Was the research design appropriate? | **Yes** | **Yes** | **Yes** | **Yes** | **Can't Tell** | **Yes** | **Yes** |
| Was the recruitment strategy appropriate? | **Can't Tell** | **Yes** | **Can't Tell** | **Can't Tell** | **Can't Tell** | **Yes** | **Yes** |
| Was the data collected in an appropriate way? | **Yes** | **Yes** | **Probably Yes** | **Probably Yes** | **Can't Tell** | **Yes** | **Yes** |
| Research- participant relationship considered? | **Can't Tell** | **Probably No** | **Probably No** | **Probably No** | **Probably No** | **N/A** | **Probably Yes** |
| Have ethical considerations been taken into consideration? | **Can't Tell** | **Yes** | **Yes** | **Yes** | **Can't Tell** | **Yes** | **Yes** |
| Was the data analysis sufficiently rigorous? | **Yes** | **Can't Tell** | **Yes** | **Yes** | **Yes** | **Probably Yes** | **Yes** |
| Is there a clear statement of findings? | **Yes** | **Probably No** | **Yes** | **Yes** | **Can't Tell** | **Yes** | **Probably Yes** |

**Supplementary Table S6:** Risk of bias summary quantitative studies

|  | Berg 2013 | Brolin 2022 | Brolin 2023 | Collister 2023 | Garbe 2021 | Milman 2019 | Mooney 2014 | Quinn 2022 | Thorpe 2008 | Wallace 2022 | Yardimci 2023 |
| --- | --- | --- | --- | --- | --- | --- | --- | --- | --- | --- | --- |
| **Appropriate Sampling?** | **Probably No** | **Probably No** | **Probably No** | **Probably No** | **Probably No** | **Probably No** | **Probably No** | **Probably No** | **No** | **No** | **Probably No** |
| **Was the attrition rate low enough to minimize ROB?** | **Probably No** | **Probably No** | **Probably No** | **Probably Yes** | **Probably Yes** | **Yes** | **Probably Yes** | **Probably Yes** | **Probably Yes** | **Probably No** | **Probably Yes** |
| **Was the instrument used in a valid and reliable manner?** | **Probably Yes** | **Yes** | **Yes** | **Probably Yes** | **Yes** | **Yes** | **Yes** | **Probably Yes** | **Probably Yes** | **Yes** | **Probably No** |
| **Was the instrument used in the intended way?** | **Yes** | **Yes** | **Yes** | **Yes** | **Yes*** | **Yes** | **Yes** | **Yes** | **Yes** | **Yes** | **Yes** |
| **Was a valid representation of the outcome used?** | **Probably Yes** | **Yes** | **Yes** | **Yes** | **Probably Yes** | **Yes** | **Yes** | **Probably Yes** | **Probably Yes** | **Yes** | **Probably Yes** |
| **Did the researchers check for understanding?** | **No** | **Probably Yes** | **Probably Yes** | **Probably Yes** | **Yes** | **Yes** | **Yes** | **Yes** | **Yes** | **Probably Yes** | **Probably No** |
| **Were the results analysed appropriately?** | **Probably Yes** | **Probably Yes** | **Probably Yes** | **Yes** | **No** | **Yes** | **Probably Yes** | **Probably Yes** | **Probably Yes** | **Probably Yes** | **Probably No** |
| **Overall Risk of Bias** | **Serious** | **Very Serious** | **Serious** | **Serious** | **Extremely Serious** | **Serious** | **Serious** | **Serious** | **Serious** | **Very Serious** | **Very Serious** |
